# Allelic variation in *Salmonella*: an underappreciated driver of adaptation and virulence

**DOI:** 10.3389/fmicb.2013.00419

**Published:** 2014-01-07

**Authors:** Min Yue, Dieter M. Schifferli

**Affiliations:** Department of Pathobiology, School of Veterinary Medicine, University of PennsylvaniaPhiladelphia, PA, USA

**Keywords:** *Salmonella*, colonization, adhesin, invasion, T3SS, SNPs

## Abstract

*Salmonella enterica* causes substantial morbidity and mortality in humans and animals. Infection and intestinal colonization by *S. enterica* require virulence factors that mediate bacterial binding and invasion of enterocytes and innate immune cells. Some *S. enterica* colonization factors and their alleles are host restricted, suggesting a potential role in regulation of host specificity. Recent data also suggest that colonization factors promote horizontal gene transfer of antimicrobial resistance genes by increasing the local density of *Salmonella* in colonized intestines. Although a profusion of genes are involved in *Salmonella* pathogenesis, the relative importance of their allelic variation has only been studied intensely in the type 1 fimbrial adhesin FimH. Although other *Salmonella* virulence factors demonstrate allelic variation, their association with specific metadata (e.g., host species, disease or carrier state, time and geographic place of isolation, antibiotic resistance profile, etc.) remains to be interrogated. To date, genome-wide association studies (GWAS) in bacteriology have been limited by the paucity of relevant metadata. In addition, due to the many variables amid metadata categories, a very large number of strains must be assessed to attain statistically significant results. However, targeted approaches in which genes of interest (e.g., virulence factors) are specifically sequenced alleviates the time-consuming and costly statistical GWAS analysis and increases statistical power, as larger numbers of strains can be screened for non-synonymous single nucleotide polymorphisms (SNPs) that are associated with available metadata. Congruence of specific allelic variants with specific metadata from strains that have a relevant clinical and epidemiological history will help to prioritize functional wet-lab and animal studies aimed at determining cause-effect relationships. Such an approach should be applicable to other pathogens that are being collected in well-curated repositories.

## ONE TO FIVE STAR HOTELS FOR *Salmonella*

*Salmonella enterica* infections result in substantial morbidity and mortality worldwide, both in humans and livestock (Hirsh, 2004). The immunopathology induced during the infectious process is the result of both *Salmonella* virulence factors and host responses (de Jong et al., 2012). Salmonellosis, when not self-limiting, requires antimicrobial therapy, particularly for neonates and to treat or prevent systemic infections. As such, the increasing prevalence of multidrug resistant *S. enterica* raises substantial concerns regarding the efficacy of current therapy. Continuous surveillance of reported *Salmonella* cases (Centers for Disease and Prevention, 2013) indicates that *Salmonella* remains the most frequent bacterial cause of foodborne disease in the US and suggests that control programs aimed at reducing food contamination have not succeeded for *Salmonella*. In this regard, the persistence of *S. enterica* in the intestinal tract in a large number of food animals creates chronic or non-symptomatic carriers that continue to shed bacteria in feces, thereby serving as a reservoir for future spread by contaminated meat, milk, eggs, and agricultural products grown on land fertilized with *Salmonella-*containing manure. The *Salmonella* reservoir is not only maintained by transmission between animals, directly or indirectly through insect vectors (Wales et al., 2010), but also by long-term environmental contaminations. Indeed, some *Salmonella* strains can survive for days, weeks, or months in the environment, and can encamp in soil, water, or on plants by making protective biofilms before being re-ingested by animals or humans. Maintenance of the environmental reservoir is enhanced by *Salmonella*’s aggressive behavior toward competitors or predators such as fungi, ameba, and helminthes, and possibly other bacteria (Aballay et al., 2000; Wildschutte and Lawrence, 2007; Tampakakis et al., 2009; Pezoa et al., 2013). Some *Salmonella* can even invade and hide inside certain plants, which if edible, add another source of transmission to humans for this successful pathogen (Arthurson et al., 2011; Golberg et al., 2011).

With the latest official publications on serological grouping of *Salmonella* up to 2007, the number of different serovars amounts to 2625 (Grimont and Weill, 2007; Guibourdenche et al., 2010). Two species are recognized, *bongori* and *enterica*, the latter species being divided in six named or numbered subspecies (*enterica* or I, *salamae* or II, *arizonae* or IIIa, *diarizonae* or IIIb, *houtenae* or IV, and *indica* or VI; V became *S. bongori*; Tindall et al., 2005). Many *Salmonella* strains survive and multiply in a range of environments, but the most “luxurious” accommodations are warm and rich in food. For example, although most *S. enterica* subspecies preferentially lodge in the intestines of cold-blooded animals, *S. enterica* subsp. *enterica* (also designated subsp. I) has “upgraded” to the bowels of some warm-blooded animals, the optimal environment for bacterial multiplication and transmission. Moreover, the maintained ability of *S. enterica* to “camp” outside of the host in less opulent telluric biotopes further improves its chances of transmission.

While many of the approximately 1,500 characterized serovars of that subspecies seem to promiscuously colonize any mammal or bird, others show adaptation to a subset of host species, or may even be restricted to one host species. *Salmonella* serovar variants with distinct host range are well documented in host species-specific endemic or epidemic strains (Rabsch et al., 2002). While comparative genomic studies of *Salmonella* strains highlight evolutionary steps involving gene gain by horizontal gene transfer (HGT), as well as recombination, mutations and gene loss (Porwollik et al., 2002; Holt et al., 2009; den Bakker et al., 2011), determining which genetic alterations directly participate in host adaptation remains a barely started puzzle. It is clear, for example, that bovines don’t offer “room and board” to the avian-restricted *S. enterica* serovar Gallinarum or the human restricted *S. enterica* serovar Typhi. However, both the bovine-adapted *S. enterica* serovar Dublin as well as serovar Typhimurium, which has a broad host range, can colonize cattle whose levels of immune competence and competing flora dictate the one to five star “quality” rating of the host environment. Serovars such as serovar Dublin that have a more defined host restriction are often more virulent toward their hosts. For example, serovar Dublin causes a potentially lethal systemic infection in its bovine host. However, since not all serovar Dublin-infected hosts die, the high level of multiplication and transmission in chronically ill hosts and/or convalescent silent carriers appears to trump the occasional death of individual hosts. In contrast, the ability of broad host range serovars to subsist in any warm-blooded animal seems to compensate for their less extensive host invasion, potentially explaining why the broad host range serovar Typhimurium remains a most isolated *Salmonella* serovar in veterinary and human medicine.

## *Salmonella* LIGAND ALLELES: THE DEVIL IS IN THE DETAILS

Current *Salmonella* genomic studies identify potential genetic evolutionary adaptations using a variety of *in silico* approaches that analyze genetic differences in collections of subspecies and serovars (Soyer et al., 2009). A recent study compared tree-building methods to ascertain congruent results by integrating models of gene gains and losses (Desai et al., 2013). Incremental information was built with strain-specific lists of new or missing groups of genes that might be involved in strain adaptation and virulence. However, to date, such studies have mainly investigated differences between serovars. Strains within a serovar are typically more related to each other, suggesting that genes responsible for the O and H antigens of *Salmonella*, which determine serovar classification, are latecomers in *Salmonella*’s evolutionary history. However, it is sometimes unclear whether the strains studied within a serovar are independent and not clonal, considering that many of the histories and pathotypes (origin, host species, disease and pathology) remain poorly defined. Thus, the observed lack of diversity within a serovar might be partially biased by the collection of strains studied.

Strain differences within the same serovar, particularly for those of broad host range, have long been under-evaluated, as exemplified by the increasing number of well-documented “exceptions” that illustrate distinct lineages within such serovars (Sangal et al., 2010; Lawson et al., 2011; Okoro et al., 2012; Gragg et al., 2013). However, it is quite likely that the ability of *Salmonella* to colonize and/or cause diseases in different hosts depends not only on the presence of a collection of specific genes, but also on the allelic variation within these genes. In surprising contrast to research in both cancer and metabolic and inheritable diseases, where mammalian single nucleotide polymorphisms (SNPs) are dissected in great detail to determine potential causality (Frazer et al., 2009), few investigators have assessed allele-mediated effects of non-synonymous SNPs on bacterial proteins that mediate or participate in host specificity and virulence (Hopkins and Threlfall, 2004). Notably, the few studies that have assessed allelic effects of virulence factors in *Salmonella* pathogenesis revealed altered virulence phenotypes (Wagner and Hensel, 2011; Thornbrough and Worley, 2012). The potential role of non-synonymous SNPs on host specificity is best exemplified with the orthologous fimbrial adhesin FimH of the avian-specific serovars Gallinarum and Pullorum which mediates significantly better bacterial binding to chicken leukocytes than did serovar Typhimurium FimH alleles (Guo et al., 2009). In contrast to the latter alleles, the avian-specific FimH did not mediate bacterial binding to mammalian cells. Even though the FimH of serovar Typhimurium mediates specific binding to mannosylated residues on glycoproteins, as expected of the adhesion of type 1 fimbriae in general, binding to chicken leukocytes by fimbriated bacteria expressing the avian-specific FimH was only minimally inhibited by mannose. Notably, FimH of serovar Gallinarum and Typhimurium differ by only 5–6 amino acids, but one amino acid substitution was sufficient to restore mannose-specific binding (Kisiela et al., 2005). Moreover, the mannose-inhibitable FimH of serovar Enteritidis which has the latter amino acid substitution, decreased chick colonization when studied in serovar Gallinarum (Kuzminska-Bajor et al., 2012). Together, these data demonstrated that allelic variation of the *Salmonella* FimH adhesin directs not only host-cell-specific recognition, but also distinctive binding to mammalian or avian receptors. Remarkably, the allele-specific binding profile parallels the host specificity of the respective FimH-expressing pathogen, highlighting its physiologic relevance.

## *Salmonella’s* SWAT TEAMS FOR HOST COLONIZATION

The ability of *Salmonella* to colonize its host(s) relies on sets of molecules that allow it to bypass natural host defense mechanisms, such as gastric acidity and gastrointestinal proteases and defensins, as well as the aggressins of the intestinal microbiome. In addition, *Salmonella* utilizes surface molecules, organelles, and machineries, such as adhesins, flagella, and type 3 secretion systems (T3SS) to actively make contact with host cells and deliver molecules that initially improves host colonization, and later its transmission to new hosts. As such, allelic variation in any of these molecules could influence host specificity and pathogenicity.

**FIGURE 1:**
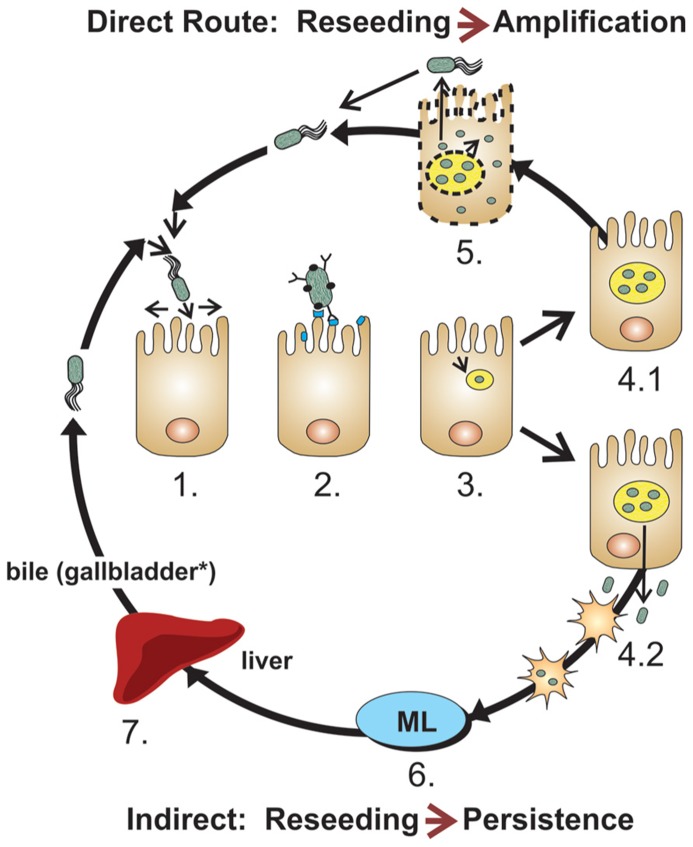
**Model of intestinal colonization by *Salmonella*.** Most *Salmonella* remain associated to specific anatomical segments of the intestines using cyclical multiplication steps in the lumen and enterocytes, using (1) near surface swimming, (2) adhesion (several adhesins), (3) invasion with formation of SCV, (4.1) intravacuolar replication, (5) escape in the cytoplasm, pyroptosis and return to the intestinal surface and lumen. After intravacuolar replication, (4.2) *Salmonella* can also escape toward the lamina propria, resulting in their potential uptake by dendritic cells and macrophages. (6) Some *Salmonella* can be further transported to the mesenteric lymph nodes which frequently contain further spreading. Typically for host-restricted (e.g., serovar Typhi in humans) or host-adapted strains (e.g., serovar Dublin in bovines), but also for *Salmonella* with broad host range encountering stressed hosts or weakened host immune responses, (7) *Salmonella* can cause bacteremia. When disseminating *Salmonella* (whether or not host-adapted or restricted) can be somewhat controlled by the host, they typically settle in the liver, gallbladder (for mammals with this organ*), on calculi and in the biliary tract, where they can replicate and be released in the bile to return to the intestines (Oboegbulem and Muogbo, 1981; Cavallini et al., 1991; Wood et al., 1991; Pellegrini-Masini et al., 2004; Akoachere et al., 2009; He et al., 2010).

Flagella direct the *Salmonella* toward the surface of intestinal epithelium by promoting bacterial movement across the mucus layer (**Figure 1**, step 1). Flagella’s near-surface swimming properties optimize the encounter of bacterial adhesins with host receptors (Humphries et al., 2001; Korhonen, 2007; Wagner and Hensel, 2011; Misselwitz et al., 2012). Flagella have highly variable alleles (which serve as the basis for H antigen serology in *Salmonella*). While flagellar motility is clearly required for intestinal colonization (Robertson et al., 2000), early studies suggested that flagella act as adhesins (Allen-Vercoe and Woodward, 1999) and flagella were reported to mediate bacterial binding for biofilm formation on cholesterol-coated surfaces (Crawford et al., 2010). Thus, in addition to providing antigenic variation to allow *Salmonella* to escape a host’s immune system, variations in *Salmonella* flagellin sequences may also influence the efficiency of intestinal colonization.

*Salmonella* encodes a large collection of adhesins, including fimbrial organelles (Yue et al., 2012a), surface associated proteins or surface-exposed domains of anchored outer membrane proteins (OMPs; Wagner and Hensel, 2011), which can attach to enterocytes (**Figure 1**, step 2), phagocytic cells (**Figure 1**, step 4.2), or gallbladder and biliary calculi (**Figure 1**, step 7; Reisner et al., 2003; Ledeboer et al., 2006; Dwyer et al., 2011; Gonzalez-Escobedo et al., 2011; Spurbeck et al., 2011; Wilksch et al., 2011). A subset of the adhesive OMPs are autotransporter proteins with surface-exposed adhesive N-terminal ends or passenger domain that have the potential to influence host-specificity (Kingsley et al., 2003, 2004; Morgan et al., 2004; Dorsey et al., 2005; Raghunathan et al., 2011; Wagner and Hensel, 2011; Leyton et al., 2012). Other OMPs have adhesive properties or significant sequence similarities to known adhesins, some which promote epithelial cell invasion by a zipper mechanism (Velge et al., 2012). *Salmonella* adhesins secreted by type 1 secretion systems (T1SS, ABC transporters) and anchored on the bacterial surface (Morgan et al., 2004; Latasa et al., 2005; Gerlach et al., 2007; Wagner and Hensel, 2011) may also participate in specific host colonization.

*Salmonella* optimizes transmission and intestinal colonization by invading and replicating in enterocytes, a process that is mediated by subversion of the enterocyte’s cytoskeletal machinery and signal transduction systems by secreted/injected effector proteins from the two T3SS (Stevens et al., 2009; Heffron et al., 2011). For this, adhesion-mediated attachment stabilizes bacterial docking by the *Salmonella*-pathogenicity island 1 (SPI-1) encoded T3SS, which then injects effector proteins into enterocytes (Misselwitz et al., 2011) to trigger bacterial uptake by macropinocytosis (**Figure 1**, step 3). Once inside the *Salmonella*-containing vacuole (SCV), *Salmonella* deploys a second T3SS encoded by SPI-2 to inject effector proteins that optimize conditions for bacterial survival and replication (**Figure 1**, step 4.1; Stevens et al., 2009; Heffron et al., 2011). Additional virulence factors subsequently allow hyper-replicating bacteria to escape the SCV, thereby promoting a cytosolic inflammatory response that results in pyroptotic cell death. Extracellular release of bacteria from dying cells potentially contributes to colonization by allowing invasion of new enterocytes (**Figure 1**, step 5; Knodler et al., 2010). In addition, T3SS effectors direct the transport of SCVs to the basolateral side of enterocytes, where released bacteria can be taken up directly by lamina propria phagocytes (Muller et al., 2012), including dendritic cells (DCs; **Figure 1**, step 4.2), a T3S-regulated process (Swart and Hensel, 2012). Finally, effectors of the two T3SS, but particularly of SPI-2 allow *Salmonella* to replicate intracellularly or bypass phagocyte-mediated killing in the lamina propria and to hitchhike inside DCs (Swart and Hensel, 2012) to seed mesenteric lymph nodes (**Figure 1**, step 6). SPI-2 effectors also interfere with MHC-presentation and activation of an immune response that would eliminate *Salmonella*. If not contained, the bacteria that escape the lymph nodes promote a primary bacteremia that seeds the liver (**Figure 1**, step 7), to initiate a circular reinfection process by taking the biliary route to return to the intestinal lumen. In addition to making biofilms on gallbladder stones, *Salmonella* adheres to and invades gallbladder epithelial cells (and probably biliary tract epithelial cells) in a SPI-1-dependent manner (Gonzalez-Escobedo and Gunn, 2013), creating a constant or recurring seeding source for the intestines. Some T3SS effectors might play several roles, such as the SPI-2 effector SseI/SrfH that was suggested to activate cell motility early during intestinal infection, but also to mainly inhibit cell migration later during infection, after establishment of new reservoirs in the mesenteric lymph nodes and liver (Worley et al., 2006; McLaughlin et al., 2009). Thus, effector proteins of the two T3SS promote various steps of intestinal, mesenteric, and hepatic survival and replication, some inducing anti-inflammatory responses, creating stable seeding reservoirs that contribute to host’s persistent colonization (Monack et al., 2004; Heffron et al., 2011; Gopinath et al., 2012; Ruby et al., 2012).

## SNPs SNAPSHOT: *Salmonella* ET AL.

As discussed earlier for the type 1 fimbrial adhesin FimH, type 3 effector proteins of *Salmonella* harbor non-synonymous SNPs that have the capacity to modulate the infectious process. For example, the Gifsy-2 phage *srfH/sseI* gene encodes two protein alleles that differentially influence the motility of intestinal CD18-expressing phagocytes upon ingestion of *S. enterica* serovar Typhimurium (Thornbrough and Worley, 2012). Migration is stimulated by the SrfH/SseI allele with a glycine at position 103, allowing it to bind to TRIP6 to promote mobility. The same effector protein with an aspartic acid at position 103 does not interact with TRIP6, and thus does not induce phagocyte migration. Interestingly, the glycine-bearing allele is found in specific Typhimurium strains that are more invasive, cause more severe diseases or associate with outbreaks. Allelic variations among effectors of *Salmonella* T3SS include the SopE Cdc42 RhoGTPase (Hopkins and Threlfall, 2004; Schlumberger and Hardt, 2005). Although existing alleles were not directly compared functionally, a substitution of residue 198, which is variable in different serovars, affects the catalytic activity of the enzyme.

In addition to the above-mentioned virulence factors of *Salmonella*, this bacterium has evolved sophisticated subsistence factors or metabolic pathways to survive and multiply during host colonization (Deatherage Kaiser et al., 2013; Steeb et al., 2013). Multi-omic data by Deatherage Kaiser et al. revealed that *Salmonella* induces an inflammatory response that alters the intestinal microbiota, thereby increasing the availability of various metabolites, including fucose, that accumulate following depletion of commensal bacteria. Moreover, *Salmonella* responds to this shift in metabolites by upregulating expression of fucose utilizing proteins. Similarly, combining proteomics, microbial genetics, competitive infections, and computational approaches Steeb et al. identified at least 31 host nutrients that increased in infected tissues and demonstrated that *Salmonella* adjusted its catabolic pathways to utilize new host nutrients. Elegant experiments also demonstrated how *Salmonella* takes advantage of its induced inflammatory environment to out-compete the intestinal microbiota with specialized respiratory pathways (Winter et al., 2010; Rivera-Chavez et al., 2013).

An important caveat concerning most of the information on *Salmonella* virulence and infection is that it is based on *in vitro* studies, carried out with a restricted number of *Salmonella* strains, and primarily assessed in mouse models of infection, with a limited amount of data gathered in farm animals, particularly cattle and chicken. We predict that host-dependent colonization is not only modulated by specific adhesin alleles (Guo et al., 2009; Kuzminska-Bajor et al., 2012), but also by non-synonymous SNPs in additional virulence factors, such as the T3SS effectors (Schlumberger et al., 2003; Hopkins and Threlfall, 2004; Raffatellu et al., 2005; Thornbrough and Worley, 2012). It is also likely that numerous additional *Salmonella* protein alleles will be identified in the near future as virulence factors senso lato, including proteins of metabolic pathways that have evolved to take advantage of unique host species properties.

Because of its importance if the fields of immunology and vaccinology, allelic variation of antigenic microbial proteins has been studied for years. The vertebrate’s immune system exerts a powerful selection on such antigens, forcing an accelerated evolutionary route for the bacteria that use these virulence tools to breach immune recognition. In contrast, allelic variation in non-antigenic virulence genes has received less attention, possibly due to the uniqueness and complexity of genotype-phenotype links for each factor. However, differential interaction of allelic variants with host molecules has been reported for a variety of bacteria, supporting the global relevance of this pathoadaptive mechanism. For example, only SpeB (streptococcal pyrogenic exotoxin B) variants that harbor an integrin-binding motif (Stockbauer et al., 1999) mediate cell rounding and detachment from culture substrates. In addition, a specific amino acid polymorphism in the CagL protein of *Helicobacter pylori* correlates significantly with a higher risk for gastric cancer and severe corpus gastritis (Yeh et al., 2011) and OmpA allelic variants of *Bacteroides vulgatus* isolates from ulcerative colitis patients mediate better bacterial adhesion to a human colon cancer cell line than do those isolated from patients with colon cancer (Sato et al., 2010). Finally, allelic polymorphism between the YopJ/YopP type 3 effector proteins of the mammalian virulent *Yersinia* species differentially affects secretion and toxicity levels in a mouse model (Brodsky and Medzhitov, 2008). Similarly, strains of *P. syringae* can express three YopJ/YopP homologous proteins with various alleles that induce host-adapted hypersensitive responses in different plants. Thus, whereas allelic variation of YopJ/YopP in *Yersinia* modulates the type of pathology in one host, allelic variation in the corresponding proteins of the plant pathogen *Pseudomonas syringae* elicit host-specific spreading of infections (Ma et al., 2006).

## FROM GENOMICS TO GWAS: THE IMPORTANCE OF BEING EARNEST WITH METADATA

Technological improvements have reduced the cost of next generation sequencing, allowing for increasingly larger-scale investigations based on tens to hundreds of *Salmonella* strains. This trove of new information has allowed extensive studies on the adaptive history of pathogens. In addition to information on gene acquisition, inactivation, or loss, comparative genomics highlights non-synonymous mutations that may alter function. Unfortunately, the number of mutations is usually too high to make firm conclusions. In contrast, genome-wide association studies (GWAS) that focus on *Salmonella* strains for which there is host and clinical information would help to better limit the number of genes likely involved in strain-specific phenotypes. Currently, GWAS are mainly used to identify disease-associated sequences within human genomes. Specifically, whole genomes of healthy and affected individuals are sequenced and compared for intra- and inter-genic SNPs that are associated with a specific disease to suggest a potential cause and effect relationship. Discovered associations are expected to support the development of new diagnostic, therapeutic, and preventive tools. Some studies focus on genomic regions already suspected to contribute to disease progression to identify a limited number of associated SNPs. Notably, GWAS have successfully identified several disease-specific SNPs (Stranger et al., 2011).

Analogous to studies on human genomes, GWAS can be applied to bacterial genomes. Assuming one has the needed metadata to separate groups of strains in an epidemiologically, clinically, or biologically relevant manner, GWAS should be able to characterize specific SNPs in strain variants that are associated with restriction to a particular host species or strain pathotype in-between and inside serovars. Moreover, by carrying out GWAS on strains with detailed host metadata, strains restricted to the same host could be directly compared, thereby reducing the confounding effects of differential host innate immune and adaptive defense responses on virulence. In addition to host specific associations, SNPs associated with ethnicity or breed, age, and sex are some additional factors that could be evaluated by GWAS. More complicated studies can compare additional groups or variables, however, statistical methods for GWAS can be plagued by having more variables than samples, the so called “curse of dimensionality” (Kelemen et al., 2009). Increasing the number of strains with useful metadata should improve the reliability of the conclusions, but large-scale investigations of hundreds of *Salmonella* genomes still remain a challenging undertaking, considering the high cost and time-consuming analysis. A palliative plan is to restrict GWAS to genes of interest, namely genes already known to play a role in virulence or multiplication (e.g., through special metabolic pathways) in at least one host species. Several technologies for targeted sequencing have been developed in recent years, and are based on DNA enrichment methods such as capture by hybridization or microfluidic PCR (Meyer et al., 2008). Incorporating sample barcoding to the latter method recently allowed us to use massive parallel sequencing for assessing allelic variation in known and predicted fimbrial adhesins of *S. enterica* serovar Newport (Yue et al., 2012b). Despite only targeting 8 adhesin genes from 48 independent strains, we identified 5–85 SNPs per gene, with 6–12 different alleles per gene. Phylogenic trees clearly separated each of five adhesins into two groups, which overlapped significantly. Most importantly, the two groups clearly differentiated strains of bovine and non-bovine origin. Even though these preliminary results utilized a relatively small number of isolates, the data were highly promising and clearly supported the idea that adhesin alleles contribute to host specificity.

It is safe to assume that multiple mutations in a variety of genes participate in the slow and progressive course of host adaptation. In addition to binding to intestinal host receptors, *Salmonella* has evolved to colonize its diverse hosts through a tailored army of virulence factors that mediate tissue invasion and subvert host immune defense mechanisms to improve short to long-term multiplication and transmission. Identification of such factors in bacterial pathogenesis studies are traditionally based on molecular Koch’s postulates that either interrogate one candidate at a time or assess libraries of random mutants of one prototypic bacterium in an animal model. However, despite the frequent insufficiency of metadata, candidate virulence genes that encode variable alleles in *Salmonella* strains for which genomic data are available can be detected by comparative *in silico* analysis. As described above, targeted sequencing of these genes, focusing particularly on the ones predicted or found experimentally to play a role in various steps of *Salmonella*-host interactions, can then be applied to strain collections that have relevant metadata to identify statistically significant associations. The detection of such associations will help to limit the number of confirmatory “wet lab” and animal experiments required to demonstrate the role of allelic variants in specific host colonization.

## HOARDING ANTIMICROBIAL RESISTANCE BY STICKING AROUND

**FIGURE 2:**
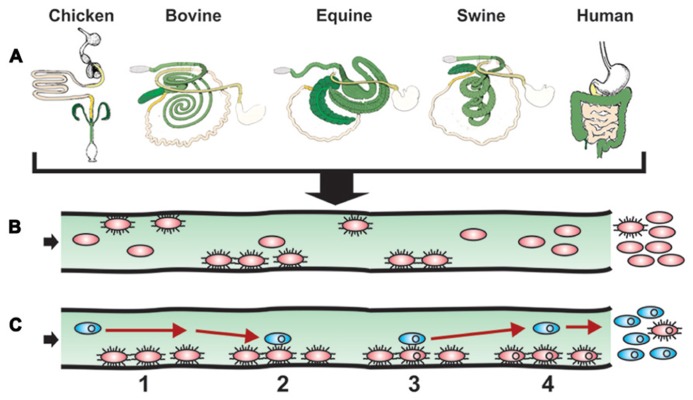
**Model of intestinal surface colonization and HGT.** **(A)** The success story of *Salmonella* evolution and adaptation to a multitude of environments and hosts is illustrated by showing how diverse the intestinal tract anatomies of just a few warm blooded animals are when comparing chicken, bovine, equine swine, and human. In addition to the distinct anatomies, histological, immunological, and microbiota particularities further participate to create unique compartments in which varieties of *Salmonella* developed ways to live and multiply, with a simple escape route for future settlements. **(B)** *S. enterica* expressing specific allelic adhesins for the cognate host intestinal receptors and cellular targets initiate successful colonization. Other strains lacking such ligands will not be retained as well in the host. A resulting gastroenteritis and/or persistence will result in the multiplication and possible long-term excretion of such *Salmonella* strains (not shown). **(C)** Intestinal colonization mediated by specific allelic adhesins of *Salmonella* (red ovals) optimizes contact (event numbers and time span) with a constant flow of new bacteria (blue ovals), some carrying antimicrobial resistance genes on conjugative or mobilizable elements (small circles in ovals), resulting in increased HGT efficiency and antibiotic-resistant *S. enteric*a colonizing carrier hosts. With time, excretion of antibiotic-resistant *S. enterica* will be excreted in greater numbers than antibiotic-susceptible *S. enterica* (not shown).

Although *Salmonella*-induced gastroenteritis is typically self-limiting, antibiotherapy is generally indicated for salmonellosis in patients or animals of young age or at risk for septicemia. As many farm animals are asymptomatic carriers of *Salmonella*, farm animals given antibiotics either routinely as food additives or therapeutically likely contribute to the alarming increase in multiple drug-resistant (MDR) *Salmonella* (McDermott, 2006)*.* The corresponding uncontrolled access to and self-prescription of oral antibiotics in the human population of developing countries and the frequently inappropriate use of antibiotics in developed countries amplify the problem. Antibiotic resistance genes in enteric bacteria, including *Salmonella*, are frequently located on mobile DNA elements such as transposons, integrons, integrative conjugative, or mobilizable elements (including conjugative transposons) and plasmids (Vo et al., 2007; Welch et al., 2007; Su et al., 2008; Ajiboye et al., 2009; Fricke et al., 2009; Lindsey et al., 2009; Call et al., 2010; Douard et al., 2010; Johnson et al., 2010; Wozniak and Waldor, 2010). HGT of these mobile DNA elements occurs in the wet and warm environment of the intestines (Nijsten et al., 1995; Lester et al., 2004; Rowe-Magnus and Mazel, 2006; Schjorring et al., 2008; Trobos et al., 2009; Faure et al., 2010), where antibiotics have been speculated to select for successful MDR gene dissemination (Beaber et al., 2004; Hastings et al., 2004). Host-specific bacterial adhesion and local invasion of the intestinal mucosa (**Figure 2A**) result in a local inflammatory response that can profit HGT events (Stecher et al., 2012). Moreover cyclical colonization of the intestines (**Figure 1**) that leads to intestinal persistence (**Figure 2B**), likely creates optimal conditions and timespans for HGT events (**Figure 2C**), thereby increasing the antibiotic resistance gene pool (further stabilized by clonal expansion and selection if antibiotics are administered). Consistent with this notion, specific colonization of the ileum can increase the rate of intra-intestinal conjugation (Garcia-Quintanilla et al., 2008). Notably, HGT is increased at high bacterial density such as in biofilms (Licht et al., 1999; Roberts et al., 2001), the generation of which is influenced by adhesins (Reisner et al., 2003; Ledeboer et al., 2006; Dwyer et al., 2011; Spurbeck et al., 2011; Wilksch et al., 2011). In addition, HGT of antibiotic resistance genes can increase expression of some adhesins (Sahly et al., 2008), suggesting a positive feedback mechanism between intestinal colonization and HGT. Interestingly, the enhanced colonization or enteritis by *Salmonella* in swine or calves, respectively (Bearson et al., 2010; Pullinger et al., 2010; Verbrugghe et al., 2012) that occurs in response to host stress (transport, feed withdrawal) is associated with enhanced intestinal CF expression and increased conjugative transfer of antibiotic-resistance plasmids (Peterson et al., 2011), further supporting a link between adhesins and HGT. As the repertoire and alleles of *Salmonella* colonization factors influence cell binding and invasion of specific hosts, one predicts that they will have a direct impact on both intestinal colonization efficiencies and the stability and rate of conjugation.

We recently tested this prediction by investigating the association of fimbrial adhesin alleles with antimicrobial resistance for eight adhesin genes from 48 strains of *S. enterica* serovar Newport (Yue et al., 2012b). Phylogenic trees clearly separated each of five adhesins into two separate groups. The allelic adhesin groups correlated significantly with strains that did or did not show antimicrobial resistance. A corresponding association was found with the presence or absence of mobile DNA elements carrying antibiotic resistance genes such as plasmids or integrons. These findings further supported the notion that some *Salmonella* adhesin alleles might participate in the persistent colonization of specific hosts and optimize the opportunity for bacterial encounters and HGT in intestines, resulting in the accumulation of antimicrobial resistance genes in such strains. Consistent with this, a new study just determined that the resistance genes of *S. enterica* serovar Typhimurium DT104 were largely maintained within animal and human populations separately (Mather et al., 2013), potentially suggesting limitations in successful transmission between host species. This novel concept deserves further investigations to confirm its biological relevance by *in vitro* and *in vivo* experiments.

## A PROMISING FUTURE, “*NOW THIS IS NOT THE END, THIS IS NOT EVEN THE BEGINNING OF THE END, BUT IT IS PERHAPS THE END OF THE BEGINNING*” (Churchill, 1943)

Future work aimed at assessing the relative importance of allelic variation in pathogenic bacteria will clearly benefit from the investigation of strains that have a complete clinical and epidemiological history. Whereas full sequencing will best serve GWAS when relevant strain metadata are available, targeted sequencing of genes of interest might be more productive by (1) permitting analysis of more strains at the same cost, (2) improving statistical power, and (3) simplifying the bioinformatics and statistical analysis. This latter approach best suits pathogens for which there are already long lists of predicted or known proteins that act as virulence factors *in vitro* or in animal models. Notably, the recent use of genomics and metabolomics have also renewed interest in bacterial metabolism by highlighting the importance of specific metabolic pathways to the intestinal, the extra-intestinal and particularly the intra-cellular lifestyle of *Salmonella* (Dandekar et al., 2012). Studies that characterized non-synonymous SNPs in metabolic enzyme genes (Allard et al., 2013), or identified distinct invasive *Salmonella* subpopulations characterized by allelic enzyme profiles (Saeed et al., 2006) strongly suggest that allelic proteins involved in *Salmonella* metabolism deserve further investigation. Similarly, allelic and phenotypic properties can be identified in regulatory proteins that participate directly or indirectly in virulence (Chen et al., 2012). Finally, while SNPs in open reading frames clearly controls the expression of allelic proteins, SNPs in cis-regulatory regions that act as operators may elicit differential recognition by downstream virulence effectors. For example, SNPs in the operator region of the *srfN* gene of *Salmonella* created a binding site for the SPI-2 regulator SsrB and a corresponding fitness gain in an oral murine model of infection (Osborne et al., 2009).

To summarize, the identification of biological consequences for allelic variants have reached the critical threshold required to stimulate more systematic investigations on well-curated strain collections, which can now exploit recently developed tools and technologies, such as GWAS and targeted sequencing.

## Conflict of Interest Statement

The authors declare that the research was conducted in the absence of any commercial or financial relationships that could be construed as a potential conflict of interest.
